# Selecting learning partners: memory for participation and competence

**DOI:** 10.1186/s41235-025-00656-z

**Published:** 2025-08-03

**Authors:** Oktay Ülker, Daniel Bodemer

**Affiliations:** Research Methods in Psychology–Technology, Learning, Collaboration, University of Duisburg-Essen, Lotharstraße 65, 47057 Duisburg, Germany

**Keywords:** Group awareness, Memory for social information, Partner modeling, Academic help-seeking, Social comparison orientation, Multinomial processing tree model

## Abstract

Remembering information about others is important but challenging in various social contexts. For instance, in long-term collaborative educational settings, students often need to choose peers for academic support. In different contexts, the selection process can depend on group awareness, i.e., the state of being informed about relevant social or cognitive characteristics of (potential) learning partners, like their participation or competence. However, selection can also depend on memory for different group awareness information on peers, which is not always accurate. An experimental study (*N* = 85) examined how type (participation vs. competence) and level (high vs. medium vs. low) of presented group awareness information influence learning partner selection in two phases (when information is present and when it is remembered). Higher levels were associated with higher selection probabilities, regardless of information type. Social comparison tendencies were associated with avoiding low participation partners. Moreover, we analyzed memory for group awareness information with multinomial processing tree model-based analyses: high and low participation levels were remembered better than medium levels, whereas high competence was remembered better than medium and low competence. Findings suggest that learners use different approach and avoidance strategies for choosing learning partners based on the type of given information.

## Significance statement

Deciding whom to ask for help is a central aspect in various contexts. For example, in schools and universities, students often need to choose peers to work together with or to seek help from. These decisions are not random; they can rely on remembering important details about their peers, such as how much classmates participated in discussions in the past or how competent they are. Our study looks at how different types of information—like participation and competence—affect who students choose to work with and especially how well they remember these details later on. We found that students were more likely to choose peers who show high levels of participation and competence. Students remembered equally well if peers participated a lot or only little in the past. Regarding competence, it was better remembered if someone demonstrated high competence in the past, whereas low competence was often forgotten.

By understanding what types of social information students remember and how this influences their choice of collaborators, we can grasp the cognitive aspects of decision making in educational settings. For instance, forgetting peers’ low competence can contribute to explaining why seeking help is not always efficient: students might often ask someone who simply cannot provide help. Understanding such patterns can help educators and psychologists provide adequate guidance for students and thus help developing skills to choose the right partners for different tasks and contexts.

## Introduction

Learners are sometimes faced with the challenge of choosing adequate learning partners from a pool of potential peers. For example, students might need to choose learning partners for group projects, self-organize exam preparation groups, or find classmates who can offer help when facing academic difficulties. In such scenarios, relying on peers when seeking sources of help (academic help-seeking) is common (Giblin et al., 2021; Giblin & Stefaniak, 2021; Knapp & Karabenick, 1988; Yang et al., 2024). Especially in university cohorts, students regularly meet and interact with the same peers, and during such interactions, they can perceive (and encode) relevant attributes of their peers, like how much they participate or how competent they are. Deciding whom to ask for help is an integral part of (successful) self-regulated help-seeking (Makara & Karabenick, 2013; Newman, 2002), however, students can sometimes select inefficient sources of help (Giblin & Stefaniak, 2021). In such help-seeking processes, both perceiving and remembering information on peers can play a crucial role.

The concept of *group awareness* (GA) refers to being informed about characteristics of (potential) group members (Janssen & Bodemer, 2013). It is typically subdivided into different types of GA information, such as behavioral GA information (e.g., participation or availability) or cognitive GA information (e.g., competence or prior knowledge; Bodemer & Dehler, 2011). In computer-supported collaborative learning, instructional tools can support learners with awareness of such information: GA tools collect, transform, and display relevant information on (potential) learning partners (Bodemer et al., 2018) and can thus support the selection of adequate learning partners. According to the expectancy-value model (Makara & Karabenick, 2013), the selection of sources of help can depend on the perceived expectation that the source will provide help (which can be linked to their participation) and the perceived quality of the help the source can offer (which can be linked to their competence). Regarding competence, students seem to consider their peers’ (perceived) abilities in learning partner selection (Putzeys et al., 2023) and being aware of others’ competences can lead to better academic help-seeking processes (Schlusche et al., 2021). Additionally, being aware of peers’ participation levels motivates students to engage more in collaborative activities (Pifarré et al., 2014). While GA refers to being consciously informed on others and thus constitutes a working memory construct, the information on learning partners can also be stored in so-called partner models in long-term memory (Schnaubert & Bodemer, 2022).

For example, when students prepare for an exam in a study group, a student might notice that his peer student (Betty) actively participates a lot and demonstrates a strong understanding of a certain learning topic, whereas another peer student (Susan) rarely participates and demonstrates only a weak understanding. When a learner has collaborated with several peers, for example over the course of a semester, remembering information on them can support decision making and judging whom to ask for help. For example, a learner might meet Betty or Susan again after some time and might need to decide whether to select them as a learning partner. Remembering that Betty demonstrated a strong understanding enables informed decisions to select her as a learning partner, while remembering that Susan demonstrated a weak understanding is important to recognize her need for support instead. Remembering peers’ past participation level helps to judge whether certain peers can be expected to provide help or not, while remembering their expertise and competence level can help to judge whether a peer is a reliable source of help. Students sometimes select their learning partners based on their prior experience with them (Putzeys et al., 2023), demonstrating that academic help-seeking and selecting adequate learning partners can depend on memory for information on peers.

Taken together, the selection of peers as help-providers or learning partners can occur while learners are aware of their peers’ participation or competence level, e.g., while a GA tool presents information on peers (i.e., during partner model formation), or when they are met again without information on them being available and when information has to be remembered (i.e., during partner model retrieval). Consequently, considering the expectancy-value model of Makara and Karabenick (2013), being aware of peers’ participation levels (perceived expectation that the source will provide help) and competence levels (perceived quality of the source) can support academic help-seeking and examining memory for such information can help to understand determinants of (un)successful academic help-seeking.

### Group awareness (tools) and learning partner selection

GA tools can support learners in finding appropriate learning partners in various contexts. For example, in the experimental study of Ollesch and colleagues (2020), participants selected groups to collaborate with on a wiki article. Participants received information on the (short-term) groups regarding their average levels of knowledge, participation, and friendliness. They found that for all types of GA information, learners preferred groups with higher levels than groups with lower levels. When selecting groups to work with on wiki articles, learners considered behavioral (participation) and cognitive (knowledge) GA information as equally important. Ollesch and colleagues (2021b, 2022) also evaluated a GA tool (*uniMatchUp!*) which allows students to assess lists of peers and get information on their participation and competence. They also compared the relevance of different GA types in the selection of learning groups, this time considering a longer usage time: here, cognitive GA information was rated as more relevant than behavioral GA information. The opposing results underline the importance of considering the context of selection when weighting different GA information.

Different e-learning platforms provide learners with knowledge- and participation-related information on peers (e.g., Shi & Cristea, 2015). In such open social learner modeling, learners are provided with different (GA) information on peers. This presentation of various GA information enables social comparisons between learners (Somyürek et al., 2020), which can—under certain circumstances—improve (individual) performance (Dijkstra & Kuyper, 2008): for example, comparing themselves with more competent others can encourage learners to reflect on their own progress and set more ambitious learning goals. While the presentation of GA information can trigger (beneficial) social comparison processes, not every person is prone to compare themselves with others to the same extent. This individual variability is captured by the concept of Social Comparison Orientation (SCO, Festinger, 1954; Gibbons & Buunk, 1999). SCO is considered a stable personality trait that describes the extent to which individuals engage in and are affected by social comparison processes in everyday life. Individuals high in SCO are more attentive to social information and more likely to compare their abilities, opinions, or performances with those of others. In learning contexts, SCO can shape how learners make use of GA information (Neugebauer et al., 2016; Ollesch et al., 2020; Ray et al., 2013). For example, Neugebauer and colleagues (2016) have shown that learners with higher SCO tend to use information more which allows for comparisons between them and others, such as GA information. Therefore, when selecting learning partners based on perceived GA information on them (such as their participation or competence), individuals high in SCO may be particularly likely to attend and utilize this information—resulting in a greater tendency to approach high-level partners and avoid low-level partners compared to individuals low in SCO.

### Context-dependent memory

Crook argues that GA is a central defining aspect of computer-supported collaborative learning and states that “previous interactions [of group members] may be salient in memory” (Crook, 2022, p. 171), underlining the importance of measuring memory for GA information. Especially in cohorts, GA information must often be remembered, for example to judge whether certain peers one regularly meets can provide help, are in need of help, or are appropriate partners for group projects. Indeed, such partner modeling processes are important for efficient collaboration and better modeling is associated with better learning outcomes (Sangin et al., 2011). Usually, in partner modeling research, researchers measure how accurate partner models are, for example, by comparing presented and remembered GA information, such as calculating the overall difference between visualized and reported knowledge levels of learning partners (e.g., Erkens & Bodemer, 2019). Separately analyzing memory for high, medium, and low levels can help to distinguish the efficiency of different social learning strategies: for example, accurately remembering high competence levels might indicate good help-seeking strategies, while memory for low competence levels is important to provide needed help for weaker students.

Research regarding memory for social information about others (in some contexts referred to as source memory, e.g., Buchner et al., 2009; Kroneisen, 2018, 2024, or reputational memory, e.g., Hechler et al., 2016) suggests that there is an adaptive memory advantage for behavior and person information that is relevant in the given context (Çavuşoğlu, 2020). For example, information that helps us avoid uncooperative persons or approach cooperative persons is better remembered than information with no informational value regarding avoiding or approaching others (Buchner et al., 2009; Hechler et al., 2016). Memory for past behavior of others is crucial because it helps to predict their future behavior (Kroneisen & Bell, 2022). Thus, memory for social information is directly related to approach and avoidance strategies (Kadwe et al., 2022, for a recent review, see Sklenar & Leshikar, 2024) and adaptive behavior: better memory for past behavior of others is associated with approaching cooperators and avoiding cheaters among them (Schaper et al., 2019). Also, certain memory advantages for behaviors of others only occur when we expect to meet them again, but not if that is unlikely (Kroneisen, 2024).

In the context of learning partner selection, remembering high levels should have an adaptive function and help students to approach peers who are good choices as learning partners and potential sources of help who are likely to provide (adequate) help. Remembering that a potential partner is associated with a low level should also have an adaptive function, as this helps students avoid persons who are unlikely to participate or help (low participation), or to judge that they might need help (low competence). Here, remembering competence and participation can have different effects: competence-related information helps to judge if someone can (not) provide help, while participation-related information informs if someone is (not) likely to provide help. Analyzing memory for different levels of different GA information can thus ultimately help to better understand different approach and avoidance strategies students use in long-term learning partner selection or help-seeking behavior.

### The present study

Learning partner selection can occur in various contexts. For example, it can occur while certain information on potential partners is present (e.g., through a GA tool) or when peers meet again and GA information has to be retrieved from memory. In our experimental study, we aim to transfer methods and paradigms used in other research areas regarding person memory to a computer-supported collaborative learning context (here: learning partner selection). Our goal is to understand the effects of different types (behavioral GA: participation vs. cognitive GA: competence) and levels (high vs. medium vs. low) of GA information on selection ratings for different partners for a group project, by particularly considering memory for such GA information. This allows to test the effects of instructional measures (i.e., application of GA tools) beyond the time of application and to test how GA information on (potential) learning partners is incorporated into long-term memory and partner models. Based on the literature on learning partner or group selection (e.g., Ollesch et al., 2020), we assume that learners are more likely to select potential learning partners with higher levels of participation or competence *(H1)*. We further exploratively analyze whether the type of partner information (participation vs. competence) influences learning partner selection. Also, we examine whether the possible effects are limited to the situation of partner model formation, in which learners are provided with GA information on potential partners, or whether they remain in a subsequent partner model retrieval phase, in which the partners are presented again, but without GA information.

Based on literature regarding memory for social information (for an overview, see Çavuşoğlu, 2020), memory is better if it has an adaptive function in the given context. Because remembering high levels helps to judge whom to approach and who is likely to help, we assume that high levels of participation (*H2a*) and competence (*H2b*) are better remembered than medium levels. On the other hand, remembering low levels helps to judge who needs help and who is less likely to help. Thus, we assume that low levels of participation (*H3a*) and competence (*H3b*) are better remembered than medium levels. We will further exploratively compare memory for high and low levels of participation and competence within both conditions (high vs. low) and between them (high participation vs. high competence; low participation vs. low competence) to gain a better understanding of specific memory differences. Also, we will exploratively examine relationships between memory accuracy and selection behavior in the partner model retrieval phase.

Further considering individual differences, we will analyze interrelationships between the tendency toward social comparisons and learning partner selection behavior. As learners with higher SCO sometimes use information more which allow comparisons between them and others (e.g., Neugebauer et al., 2016), one could expect that higher SCO is associated with higher probabilities of selecting learning partners with high participation (*H4a*) and competence (*H4b*), and in line with this that higher SCO is associated with lower probabilities of selecting learning partners with low participation (*H5a*) or competence (*H5b*). We will also exploratively analyze the relationship between SCO and the selection of medium level partners and relationships between SCO and partner selection behavior in a subsequent partner model retrieval phase, in which GA information on partners is not present and has to be retrieved from memory.

## Method

### Participants

After excluding data of two participants because of technical difficulties, the final sample consisted of *N* = 85 participants (56 female and 29 male), with their ages ranging from 17 to 33 (*M* = 22.89, *SD* = 3.44). Participants were mainly university students (84.71%) and we assigned them randomly to the two conditions *participation* (*n*_1_ = 42) and *competence* (*n*_2_ = 43). The study was approved by the local ethics committee.

### Design

We used a mixed-factorial 2 × 3 design with the between-subjects factor GA type (participation vs. competence) and the within-subjects factor GA level (high vs. medium vs. low). The main phases of the experiment were the partner model formation phase, in which the participants learned about the potential learning partners with GA information on them, and the partner model retrieval phase, in which the partners were presented again, but without GA information. Central dependent variables to test our hypotheses were the partner selection ratings, which were measured in both phases, and parameter estimates of memory for GA information, which will be described in the section “Statistical analyses and (memory) measures”.

### Material and procedure

**Fig. 1:**
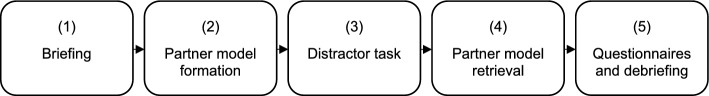
Schematic illustration of the key phases of the experiment

**Fig. 2:**
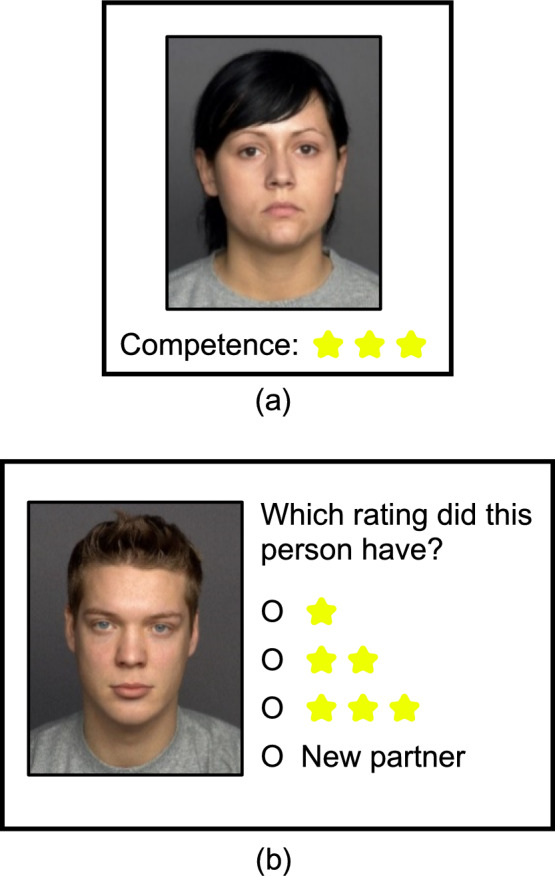
Examples for trials in the partner model formation (**a**) and partner model retrieval phases (**b**)

The experiment consisted of five main phases (see Figs. 1 and 2).

#### Briefing

Participants were informed that they would see pictures of potential (bogus) learning partners for a future group project with information on the persons. The instructions included that these persons (allegedly) worked in different group projects in the past and were rated by their partners regarding their participation or competence. This information would now be represented in form of star ratings: three stars = high, two stars = medium, and one star = low level of participation/competence. Participants were only informed that they would have to indicate their likeability and how likely it is that they would select the potential partners for a future group project. We concealed that the GA level had to be remembered in a later partner model retrieval phase.

#### Partner model formation phase

We selected 54 pictures of young adult faces (27 male and 27 female) with neutral facial expressions (Fig. 2) from the FACES database (Ebner et al., 2010). We decided for neutral facial expressions because other facial expressions can alter memory for behavior descriptions of these persons (Bell et al., 2012). Participants each saw 36 randomly chosen pictures (18 female and 18 male) in the partner model formation phase. Each trial had the same structure: first, the picture appeared. After 2 s, the text “Participation:” or “Competence:” with three, two, or one star(s) appeared below the picture (Fig. 2a), representing the GA level (high, medium, and low). The pictures were randomly and evenly assigned to the three levels (12 partners per level: six female and six male). Next, after 1 s, two scales appeared: participants first had to rate the likeability of that person on a 7-point scale from −3 (very unlikeable) to 3 (very likeable). After this, participants had to indicate how likely they were to select this person as a learning partner on a 7-point scale from −3 (very unlikely) to 3 (very likely). For the sake of parsimony, however, the likeability ratings are not reported and only the selection ratings, on which the hypotheses are based, are analyzed. After submitting the rating and clicking the “Next” button, the page cleared, and the next trial started. After 36 trials, the partner model formation phase ended. We decided to quantify the levels in a trisected manner because this can be found in literature examining the effects of GA information on learning partner or group selection (e.g., Ollesch et al., 2020, 2021a, 2021b; Schlusche et al., 2019) and in research regarding memory for social information (e.g., Buchner et al., 2009; Hechler et al., 2016; Pandeirada et al., 2017).

#### Distractor task

Participants then had to rate how much attention they paid to the star ratings on a scale from −3 (“I did not pay attention at all”) to 3 (“I paid a lot of attention”) (*M* = 1.46, *SD* = 1.49). In a distractor task, participants rated 20 mathematical equations as true or false.

#### Partner model retrieval phase

Next, the 36 previously presented partners (i.e., those from the partner model formation phase) appeared intermixed with 18 new partners (nine female and nine male) who had not been presented before. In these serial trials, the partners appeared without stars depicting their GA level. Participants again had to rate the likeability and partner selection on the same scales as in the partner model formation phase. Additionally, this time they had to indicate whether the partner was presented before in the partner model formation phase with three, two, or one star(s), or whether the partner was new (Fig. 2b). After 56 trials in a random order, the partner model retrieval phase ended.

#### Questionnaires and debriefing

Following that, participants provided metacognitive judgements. On 7-point scales from −3 (“I did not remember at all”) to 3 (“I remembered very well”), participants judged their old-new-recognition of the partners (*M* = 0.69, *SD* = 1.57) and their memory for the stars depicting the GA level (*M* = −1.62, *SD* = 1.20). Next, to assess SCO, we used the validated German version of the Iowa-Netherlands Comparison Orientation Measure (Jonas & Huguet, 2008), which consists of 11 items (e.g., “I always pay a lot of attention to how I do things compared with how others do things”) answered on a 7-point scale (Cronbach’s α =.82, *M* = 0.98, *SD* = 0.86). Additionally, social comparison motives were assessed by the Strategic Social Comparison Motives Measure (Ray et al., 2017). However, for the sake of parsimony, only the findings regarding SCO will be reported.^1^ Afterwards, participants indicated on 7-point scales from 0 (“I do not agree at all”) to 6 (“I fully agree”) whether they thought the presented persons were real potential partners (*M* = 2.01, *SD* = 1.57) and whether the stars truly indicated their participation/competence based on their past group projects (*M* = 2.48, *SD* = 1.88). Eventually, participants provided demographic information (age, gender, and education) and were debriefed. The experiment lasted approximately 30 min.

### Statistical analyses and (memory) measures

Central dependent variables for our hypotheses regarding learning partner selection (*H1*, *H4,* and *H5)* were the partner selection ratings. For each participant, we averaged partner selection ratings for the 12 learning partners per GA level for each phase of the experiment. We analyzed *H1* with mixed-factorial ANOVAs and *H4* and *H5* with regression-based analyses.

**Fig. 3:**
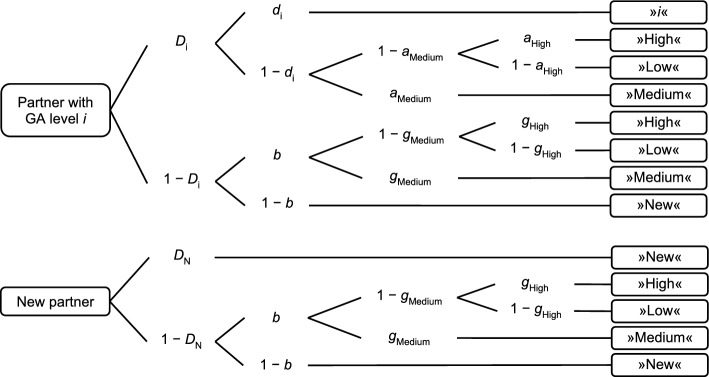
Multinomial processing tree model. The multinomial processing tree model of source monitoring (Bayen et al., 1996) for three sources (Keefe et al., 2002) was adapted for the present study purposes. GA = group awareness. Rectangles on the left side represent types of presented partners in the model formation phase. Index *i* denotes the level of GA information, *i* ∈ {high, medium, and low}. Rectangles on the right side represent the possible answers of participants in the memory test. Letters along the links represent probabilities of certain cognitive processes. *D* = probabilities of recognizing a presented partner as previously presented (*D*_i_) or as new (*D*_N_). *d* = probability of remembering the previous GA level of a presented partner. *b* = probability of guessing that a partner has been presented before. *a*_Medium_ = probability of guessing that a recognized partner was associated with a medium level. *a*_High_ = probability of guessing that a recognized partner was associated with a high level, given it was not guessed that the partner was associated with a medium level. *g*_Medium/High_ = probabilities of guessing the GA level, given the partner was not recognized as previously presented

To analyze memory for GA information (*H2* and *H3*), we used two measures, which were both based on observed classifications in the partner model retrieval phase (see Appendix A, Table 4). (1) We calculated classification-based measures for the separate levels (high, medium, low) by dividing the number of correct responses in the partner model retrieval phase by the sum of “high”, “medium”, and “low” responses for the same level (for more specific examples, see Appendix A). In source memory research, these measures are referred to as Conditional Source Identification Measures (Bröder & Meiser, 2007; Murnane & Bayen, 1996), which will be called Conditional Level Identification Measures (CLIM) here. Such approaches were used previously in different studies examining memory for information on other persons (e.g., Kadwe et al., 2022; Urban Levy et al., 2023). (2) We also used the two-high threshold multinomial processing tree (MPT) model of source monitoring (Bayen et al., 1996) for three sources (Keefe et al., 2002), adapted for our study purposes (Fig. 3). These models have been proven useful to accurately measure (person-)memory differences unconfounded by (often found) guessing biases (Bröder & Meiser, 2007; for applications, see, for example, Buchner et al., 2009; Hechler et al., 2016). For a review of MPT models, see Erdfelder and colleagues (2009). Further descriptions of the applied MPT model in particular can be found in Appendix A. MPT model-based analyses were conducted with multiTree (Moshagen, 2010) and are based on the goodness-of-fit statistic *G*^2^ (Hu & Batchelder, 1994).

Both measures can complement each other’s weaknesses: while the first measure (CLIM) can sometimes confound memory and guessing (Bröder & Meiser, 2007), it is estimated for individual participants and thus, general linear model-based regression analyses can be applied. While MPT model-based analyses estimate memory parameters unconfounded by guessing processes (Erdfelder et al., 2009), one disadvantage is that (here) it is estimated on group levels and individual differences cannot be considered.^2^

Correspondingly, we used CLIM as a classification-based measure to exploratively analyze relationships with partner selection behavior in the partner model retrieval phase in which learning partners were presented without GA information. For specific memory differences (and thus to test *H2* and *H3*), however, we used the parameter estimates of our joint MPT model.

Power analyses with G*Power (Faul et al., 2007) revealed that given *N*_Participation_ = 2268 (42 participants × 54 items in the partner model retrieval phase), *N*_Competence_ = 2322 (43 participants × 54 items), *df* = 1 (to test *H2a*, *H2b*, *H3a*, and *H3b*), and α =.05 (for all analyses), we could detect effects of the size *w* =.06 (which translates to a difference in our MPT model-based memory parameters of Δ*d* =.28) in the MPT model-based analyses regarding memory for GA information with a statistical power of 1 − β =.80.

## Results

The following results section is structured according to the independent variables and hypotheses. The procedures outlined earlier were applied (see “Statistical analyses and (memory) measures”). Correction procedures are reported where necessary, such as degrees of freedom adjusted using a Greenhouse–Geisser correction or alpha levels corrected by Bonferroni-Holm.

### Learning partner selection

To test our first hypothesis that learners are more likely to select learning partners with higher levels of participation or competence (*H1)*, we analyzed whether learners preferred to select potential learning partners with higher participation or competence levels. We therefore calculated a 2 (GA type: participation vs. competence) × 3 (GA level: high vs. medium vs. low) mixed-factorial ANOVA for the partner selection ratings in the partner model formation phase. We corrected degrees of freedom by Greenhouse–Geisser because of violations of sphericity assumptions, (Mauchly’s) *W* =.46, *p* <.001. Table 1 provides descriptive data. The ANOVA revealed a significant and large main effect of GA level on learning partner selection, *F*(1.30, 107.74) = 251.46, *p* <.001, η_p_^2^ =.75. High-level labeled partners received higher selection ratings than medium-level labeled partners, *t*(83) = 10.76, *p*_Holm_ <.001, and medium-level labeled partners received higher selection ratings than low-level labeled partners, *t*(83) = 15.84, *p*_Holm_ <.001. This supports *H1* and indicates that participants attended GA information, which is a prerequisite for further memory processes. There was neither a significant main effect of GA type, *F*(1, 83) = 0.47, *p* =.493, η_p_^2^ =.01, nor a significant interaction between GA type and GA level, *F*(1.30, 107.74) = 1.06, *p* =.325, η_p_^2^ =.01. Further exploratory examinations of simple main effects revealed that for all GA levels, partner selection did not differ between the participation and competence conditions, *t*(83) ≤ 1.44, *p* ≥.154.

**Table 1:** Mean partner selection ratings as a function of experimental phase, group awareness type, and level

Phase	Group awareness type	Group awareness level			
		High	Medium	Low	New partners
		*M* (*SEM*)	*M* (*SEM*)	*M* (*SEM*)	*M* (*SEM*)
Partner model formation	Participation	1.53 (0.15)	0.59 (0.11)	−1.48 (0.15)	-
	Competence	1.46 (0.13)	0.60 (0.11)	−1.19 (0.14)	-
Partner model retrieval	Participation	0.34 (0.09)	0.14 (0.11)	−0.31 (0.09)	−0.00 (0.10)
	Competence	0.35 (0.11)	0.29 (0.10)	0.02 (0.11)	−0.01 (0.10)

Values in parentheses represent standard errors of the means. The selection ratings ranged from −3 (very unlikely to select this person) to 3 (very likely to select this person). In the partner model formation phase, the participation or competence level of the potential learning partner was presented, while this information was absent in the partner model retrieval phase

For the selection ratings in the partner model retrieval phase, sphericity was not violated, (Mauchly’s) *W* =.98, *p* =.465. The ANOVA again revealed a significant and large effect of GA level, *F*(2, 166) = 25.03, *p* <.001, η_p_^2^ =.23, which was, however, less pronounced than in the partner model formation phase. Partners who were previously associated with low levels received lower selection ratings than partners who were previously associated with medium levels, *t*(83) = 4.75, *p*_Holm_ <.001, or high levels, *t*(83) = 7.23, *p*_Holm_ <.001. The difference between medium- and high-level partners, however, was not significant, *t*(83) = 1.80, *p*_Holm_ =.075. Again, there was no significant effect of GA type, *F*(1, 83) = 1.87, *p* =.176, η_p_^2^ =.02, and no significant interaction effect between GA type and GA level, *F*(2, 166) = 2.62, *p* =.076, η_p_^2^ =.03. We calculated simple main effects to test exploratively whether there were differences between the GA types for separate GA levels. There were no significant differences for high GA levels, *t*(83) = 0.04, *p* =.965, and medium GA levels, *t*(83) = 1.04, *p* =.300. However, partners with low participation received lower selection ratings than partners with low competence, *t*(83) = 2.23, *p* =.028. Note, however, that this comparison is no longer significant if tested against Bonferroni-Holm adjusted α =.017. Overall, the results suggest that participants partially remembered GA information and based their selection behavior in the partner model retrieval phase on their memory traces, which will be tested in the next section.

### Memory for group awareness information

**Fig. 4:**
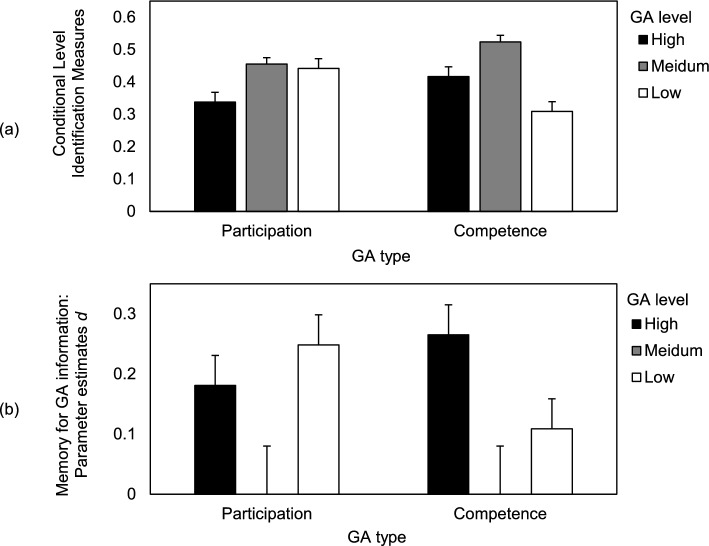
Memory for group awareness information. GA = group awareness. The error bars reflect standard errors of the means. **a** Conditional level identification measures reflect the ratio of correct to incorrect GA level judgements for learning partners judged as previously presented. **b** Parameter estimates *d*: The probability of correctly remembering the level of participation/competence of a presented partner in the partner model retrieval phase. Parameters *d* are based on the multinomial processing tree model-based analyses. Both measures can range between 0 and 1

We further tested whether CLIM (for descriptives, see Fig. 4a) as classification-based measures predicted the selection behavior in the partner model retrieval phase when participants had to retrieve information on their potential partners from memory (Table 2 presents overall model tests). For previously high-level labeled partners, CLIM_High_ significantly predicted the selection ratings, both regarding participation-related information (*b* = 1.00, β =.34) and competence-related information (*b* = 1.73, β =.47): better classifications (potentially due to better memory) were associated with higher selection ratings. However, CLIM_Medium_ did not significantly predict selection ratings, neither in the participation condition (*b* = 1.00, β =.23), nor in the competence condition (*b* = 1.02, β =.24). CLIM_Low_, on the other hand, predicted significantly and negatively selection ratings: better classifications were associated with lower selection ratings for potential learning partners previously rated with low participation (*b* = −0.94, β = −.31) or low competence (*b* = −2.31, β = −.49). It is important to highlight that these relationships need to be interpreted with caution because CLIM can confound memory and guessing processes (Bröder & Meiser, 2007).

**Table 2:** Linear regression models for CLIM (predictor) and partner selection ratings (outcome variable)

	Group awareness level		
Group awareness type	High	Medium	Low
Participation	*F*(1, 41) = 5.27, *p* =.027, *R*^2^ =.11	*F*(1, 41) = 2.31, *p* =.136, *R*^2^ =.05	*F*(1, 41) = 4.33, *p* =.044, *R*^2^ =.10
Competence	*F*(1, 40) = 11.38, *p* =.002, *R*^2^ =.22	*F*(1, 40) = 2.35, *p* =.133, *R*^2^ =.06	*F*(1, 41) = 12.86, *p* <.001, *R*^2^ =.24

CLIM = Conditional Level Identification Measure. The selection ratings were the ratings from the partner model retrieval phase, in which information on potential learning partners were not present and had to be remembered

We additionally analyzed memory for GA information with MPT models. To obtain an identifiable base model, a standard assumption of the two-high threshold model is that (at least) one *D*_Old_ parameter must be set equal with the *D*_New_ parameter (Bayen et al., 1996). We set the parameters *D*_Medium_ = *D*_New_ equal in both experimental conditions, which fits our data well, *G*^2^(2) = 4.61, *p* =.100. For the sake of completeness, all parameter estimates are depicted in Appendix A (Table 5). Our hypotheses, however, are based on the *d*-parameters (*d*_High_, *d*_Medium_, and *d*_Low_), which here reflect memory for GA level (i.e., the probability of remembering the specific GA level) and are depicted in Fig. 4b.

Regarding participation, to test whether memory for high participation is better than memory for medium participation (*H2a*), we set the parameters *d*_High_ and *d*_Medium_ equal in the participation condition. This restriction resulted in a significant reduction in the model fit, Δ*G*^2^(1) = 5.78, *p* =.016, *w* =.04: as expected, memory for high participation was better than memory for medium participation. There was also a memory advantage for low participation compared to medium participation, Δ*G*^2^(1) = 10.04, *p* =.002, *w* =.05, which supports *H3a*. Exploratively analyzing the difference between high and low participation showed no significant difference, Δ*G*^2^(1) = 0.97, *p* =.325, *w* =.01: low participation was only descriptively better remembered than high participation.

Regarding competence, in line with *H2b*, memory for high competence was better than memory for medium competence, Δ*G*^2^(1) = 7.84, *p* =.005, *w* =.04. In contrast to memory for participation and contradicting *H3b*, memory for low competence did not differ from memory for medium competence, Δ*G*^2^(1) = 1.38, *p* =.241, *w* =.02. Explorative analyses showed that high competence was also better remembered than low competence, Δ*G*^2^(1) = 6.05, *p* =.014, *w* =.04.

We exploratively compared memory for high and low levels between the different GA conditions. High participation and competence were equally well remembered, Δ*G*^2^(1) = 1.86, *p* =.172, *w* =.02, while memory for low participation was better than memory for low competence, Δ*G*^2^(1) = 4.69, *p* =.030, *w* =.03.

### Social comparison orientation and partner selection

We additionally tested for relationships between SCO and the selection ratings for partners with high, medium, or low levels of participation or competence in the partner model formation phase (in which the GA information was present) with linear regressions. While Table 3 presents overall model tests (i.e., *F*-values), these are not suitable for testing our directional hypotheses. Therefore, for the hypothesis-related analyses concerning SCO × selection of high- and low-level labeled partners in the partner model formation phase, we will analyze and interpret the regression coefficients and their associated *t*-tests. Given our directional hypotheses, we report one-tailed *p*-values by dividing the standard (two-tailed) *p*-values by two. For exploratory analyses, we rely on the overall model tests (*F*-values), as no specific directional hypotheses were formulated in these cases.

**Table 3:** Linear regression models for social comparison orientation (predictor) and partner selection ratings (outcome variable)

	Group awareness level		
Group awareness type	High	Medium	Low
	*Partner model formation phase*		
Participation	*F*(1, 41) = 2.80, *p* =.102, *R*^2^ =.06	*F*(1, 41) = 0.67, *p* =.419, *R*^2^ =.02	*F*(1, 41) = 3.77, *p* =.059, *R*^2^ =.08
Competence	*F*(1, 40) = 0.33, *p* =.572, *R*^2^ =.01	*F*(1, 40) = 0.48, *p* =.490, *R*^2^ =.01	*F*(1, 40) = 0.68, *p* =.413, *R*^2^ =.02
	*Partner model retrieval phase*		
Participation	*F*(1, 41) = 0.03, *p* =.863, *R*^2^ <.01	*F*(1, 41) = 0.03, *p* =.870, *R*^2^ <.01	*F*(1, 41) < 0.01, *p* =.947, *R*^2^ <.01
Competence	*F*(1, 40) < 0.01, *p* =.998, *R*^2^ <.01	*F*(1, 40) = 0.98, *p* =.328, *R*^2^ =.02	*F*(1, 40) = 0.20, *p* =.658, *R*^2^ <.01

For high and low levels in the partner model formation phase, we refer to the main text which report one-tailed *p*-values of the associated *t*-tests, as we proposed directional hypotheses for these relationships

Contrary to *H4*, SCO did not significantly predict the selection of high-level labeled partners: neither for participation-related information, *t*(41) = 1.67, *p* =.051 (one-tailed), *b* = 0.24, β =.25, nor for competence-related information, *t*(40) = 0.57, *p* = .286 (one-tailed), *b* = 0.08, β =.09, but the relationships were descriptively positive as expected. *H5*, however, was partially supported: for participation, SCO negatively predicted the selection of low-level labeled partners (*H5a*),* t*(41) = 1.94, *p* =.030 (one-tailed), *b* = −0.28, β = −.29, but not the selection of partners with low competence (*H5b*),* t*(40) = 0.83, *p* = .207 (one-tailed), *b* = −0.11, β = −.13. Explorative linear regressions revealed no relationships between SCO and the selection of medium-level labeled partners (participation: *b* = 0.16, β =.13, competence: *b* = −0.12, β = −.11).

Further, explorative linear regressions for partner selection in the partner model retrieval phase (when the GA information had to be remembered) revealed no significant predictions of SCO, neither for participation (high: *b* = 0.04, β =.03; medium: *b* = −0.03, β = −.03; low: *b* = −0.02, β = −.01), nor for competence (high: *b* = −0.00, β = −.00; medium: *b* = −0.18, β = −.15; low: *b* = −0.07, β = −.07).

## Discussion

Academic help-seeking is a social strategy that improves learners’ academic performance (Martín-Arbós et al., 2021) by selecting appropriate sources of help or learning partners. Different applications can support students in such processes by providing *group awareness* (GA) information on (potential) learning partners. Our study explored how the type (behavioral: participation vs. cognitive: competence) and level (high vs. medium vs. low) of presented information affects partner selection in the moment GA information is present (during partner model formation) and in retrospect, when GA information on potential learning partners has to be remembered (during partner model retrieval). Moreover, we analyzed memory for GA information.

Analyses revealed that participation or competence levels of potential learning partners affected partner selection as expected (*H1*): potential partners with higher levels received higher selection ratings. When GA information was present, GA type did not affect partner selection. However, when GA information was not present anymore and had to be retrieved from memory, there were two main differences. (1) Learners were still more inclined to select learning partners with higher levels; however, the selection tendencies did not differ between partners with high and medium levels. (2) Learners were less likely to select partners with low participation than partners with low competence. There were no differences between participation and competence regarding the selection of medium or high-level partners. Note, however, that the difference between the selection of low participation and low competence partners did not hold against Bonferroni-Holm adjustment and should thus be interpreted with caution.

Further, classification-based analyses of conditional level identification measures (CLIM) have shown that learners’ tendency to select learning partners in contexts where information on potential learning partners was not externally represented anymore may be functionally dependent on memory: students who better remembered that certain previous partners were associated with high participation or competence showed higher tendencies to select those partners. Similarly, students who better remembered that certain previous partners were associated with low participation or competence were less inclined to choose those partners. The findings further support the idea that memory is adaptive and predicts (future) decision making (see also Sklenar & Leshikar, 2024), transferring results from different social contexts, such as the Prisoner’s Dilemma Game (Schaper et al., 2019) or the Dictator Game (Murty et al., 2016), to learning partner selection in (computer-supported) collaborative learning. Our findings further emphasize the crucial role of memory in academic help-seeking. Note, however, that CLIM can confound memory and guessing processes (Bröder & Meiser, 2007), which is why these relationships should be interpreted with caution.

Considering memory for information about other persons, our findings give further insights about how volatile GA information is integrated into partner models (i.e., mental models about learning partners; Dillenbourg et al., 2016; Schnaubert & Bodemer, 2022) in long-term memory. Remembering high and low participation was better than remembering medium participation (which supports *H2a* and *H3a*). When remembering (past) participation of potential learning partners, it seems to be equally important to remember who participated a lot, potentially to approach these partners and because it can be expected that they provide help. Interestingly, when remembering competence, there was only a memory advantage for high competence (which supports *H2b*), but not for low competence (which contradicts *H3b*). Approaching learning partners who are competent and potentially good sources of help seems to be more important than avoiding those who are not as competent. Remembering high competence can support academic help-seeking processes in university cohorts, where students meet the same peers regularly and need to decide whether to ask certain peers for help or not. However, forgetting low competence might have undesired effects: learners might often forget that certain peers are in need of support and might thus overestimate them, which can lead to impaired learning of less competent learners (Wittwer et al., 2008).

*Social comparison orientation* (SCO) seems to affect learning partner selection under certain circumstances. Relationships between personal characteristics and selection behavior were not present when information on other students has to be retrieved from memory. However, with present GA information, we observed that SCO does not affect the selection of potential learning partners with high participation (contradicting *H4a*), but students with higher SCO tend to avoid partners with low participation more (supporting *H5a*). By contrast, regarding competence-related information, SCO does not affect selection at all (contradicting *H4b* and *H5b*). The missing effects in the competence condition are in line with the study of Ray and colleagues (2017): in their study, learners should imagine selecting a learning partner out of a pool with potential partners whose past examination scores were shown, with some of them having better or worse scores than the participants. There, SCO did not predict learning partner choice. Taking the results together, SCO does not influence the selection of potential learning partners with high or low (cognitive) attributes such as their competence or past examination scores; however, students with higher SCO seek to avoid low participating partners in particular. Further building on the idea of Ollesch and colleagues (2020), our results support that in the design of GA tools in learning partner selection, personal characteristics could be considered: students with higher SCO make use of behavioral GA information. Thus, especially for them, GA tools could contain behavioral information to fulfill their specific needs.

From different perspectives, our findings suggest that learners are concerned with avoiding free-riders or social loafers as their learning partners: while memory for high participation and high competence did not differ, there was, however, a memory advantage for low participation compared to low competence. Also, when selecting partners while information had to be retrieved from memory, low participating partners were less likely to be selected than partners with low competence. Especially learners with higher SCO were more concerned about avoiding low participating partners. Our results can be explained by low participation being often associated with free-riding behavior, which can be frustrating for other learners (Ward-Smith et al., 2010).

Following the interpretation of our hypothesis-related and exploratory results, the question arises as to which additional factors can influence learning partner selection tendencies and memory for information on (potential) learning partners. Regarding selection, aspects such as the combined or isolated presentation of GA information and the source of information can play a significant role. (1) In our study, each participant received information on either the participation *or* competence level of their partners. Ollesch and colleagues (2021b) have shown, however, that especially the combined presentation of behavioral *and* cognitive information has a positive effect on the article outcome in a wiki learning environment. This is supported by a recent meta-analysis on GA tools which has shown that combined presentations of different GA information (such as competence and participation) better support learners than individual presentations (Chen et al., 2024). (2) Additionally, the source of such GA information might play a crucial role: for example, in the study of Ray and colleagues (2017), the information on the learning partners was the scores in a past examination, whereas in our study, the learning partners were (allegedly) rated by former group members. Indeed, the source of GA information can influence the credibility of such information (Freund et al., 2025). Thus, systematically varying the presentation of GA information (e.g., combined vs. isolated) and the source of GA information (e.g., self-reports vs. ratings of others) can help to further disentangle effects of different kinds of GA information on learning partner selection, its relationships with SCO, or effects on memory for long-term learning partner selection and academic help-seeking.

Regarding memory for different levels of social information, in a recent experiment, Kroneisen (2024) observed that when selecting classmates for a group project, memory for positive and neutral behavior descriptions was the same, whereas negative behavior descriptions were better remembered. However, in our experiment, positive information (high levels) was better remembered than neutral information (medium levels) and only for participation, negative information (low levels) was better remembered than neutral information. One potential explanation for the different results between the studies is that in her study, participants were instructed to “choose group members well in order to avoid annoyance, additional work or bad grade” (Kroneisen, 2024, p. 123), which potentially lets participants focus on avoidance strategies and renders remembering negative information as a more useful strategy, further supporting the idea of memory advantages for social information (personally) relevant in a given context (Çavuşoğlu, 2020; Kroneisen, 2018). Consequently, memory patterns could differ depending on the context in which information on others has to be remembered, as well as the characteristics—such as the competence level—of the individual who has to remember the information. (1) The motivation for partner selection can have a significant effect on partner selection. For example, Ollesch and colleagues (2021a) have shown that when learners in wiki environments supported by GA tools have the goal to support others, they are more likely to select threads with low average group knowledge than threads with high average group knowledge. This pattern is reversed for learners with the goal to learn new content. Such motivations and different learning contexts can potentially also affect memory (Kroneisen, 2024). Importantly, such motivational states may not only be externally induced (e.g., via task framing), but also stem from learners’ own competence. For instance, less proficient students—who are more dependent on receiving help—may be more motivated to pay attention to and remember peers who appear competent and helpful. This idea aligns with the notion that memory is shaped by goal relevance (Çavuşoğlu, 2020; Kroneisen, 2024). Thus, remembering high-competence peers might be particularly pronounced among those who need support the most. (2) While memory for information on peers in learning partner selection and academic help-seeking has been a focal point, it is important to acknowledge that remembering information on others plays a crucial role in other contexts as well: not only help-seekers, but also teachers are confronted with having to memorize information on many students. However, we cannot conclude if teachers in classroom settings also forget low competence levels like students when they select learning partners. Güzel and Başokçu (2023) have shown that teachers of low-performance classes are less accurate in estimating students’ knowledge than teachers of high-performance classes, indicating that teachers might also be prone to forget students’ low competence. Gaining a deeper understanding of the factors affecting memory for information on other learners can help to tailor instructions (e.g., attentional guidance to low levels) based on the given context and for special recipients (e.g., for help-seekers vs. teachers).

Furthermore, in practice, days or even weeks can pass between perceiving relevant characteristics of peers and seeing those peers again. Thus, the question arises whether the observed memory performance can be transferred to a real long-term collaborative setting. However, Buchner and colleagues (2009) have shown that, albeit weaker, the same pattern of results (memory advantage for untrustworthy behavior) persists even when the retention interval was increased from several minutes to 1 week. Nevertheless, Chen and colleagues (2024) have also shown that higher intervention durations of GA tools had the largest effect (on different outcome variables). This might be linked to the fact that students forget information on their peers over the course of time. Our study demonstrates through different memory biases that longer support of GA tools might especially be relevant when help-providers use these tools because, compared to high competence, learners forget low competence more often.

From a methodological perspective, our study demonstrated the application of multinomial processing tree (MPT) models in measuring memory for social information in learning partner selection. These models could also be used to analyze memory not only in research regarding GA in partner selection, but also in different areas where the link between persons and information is crucial. The applied MPT model has been developed for source memory research in particular (remembering the person who told the information, Johnson et al., 1993), see, for example, Bayen and colleagues (1996), Symeonidou and Kuhlmann (2021), Ülker and Bodemer (2024). Applications in partner modeling research to measure memory for specific knowledge levels of learning partners regarding certain learning topics are also possible (for a recent example, see Ülker & Bodemer, 2023). This holds the potential to better assess partner modeling processes in different (computer-supported) collaborative learning contexts, without the influence of confounding guessing processes.

## Conclusion

Considering memory processes for information on others can reveal the effectiveness of certain social strategies in the long-term. For example, our findings show that memorizing high competence seems to be easier than memorizing low competence. This is beneficial for help-seekers, as this way, when students meet high competent peers repeatedly (e.g., in university cohorts), they know whom to ask for more help. However, especially help-providers (such as more knowledgeable students) depend on remembering the low competence of others to provide adequate help. Thus, while help-seeking students might gradually depend less on GA tools as they construct knowledge of competent peers, students who are asked for help and willing to provide it might depend more on GA tools representing the low competence or knowledge gaps of their peers, since remembering low competence appears to be difficult. Consequently, especially help-providing students might need more instructions guiding their attention, to consistently identify those who struggle and so that learners with low competence receive the targeted support they need.

Taken together, this study can contribute to further disentangle the effects of different types of GA information in help-seeking. Moreover, its results, based on complementary (analysis) methods, suggest that accurately considering memory processes in future studies can reveal approach and avoidance strategies that students apply when selecting learning partners and searching for help. In the end, both can help to better estimate the appropriate instructional design for short- and long-term use of GA information and find a better match of learning scenarios, students’ needs, and tool support.

## Data Availability

The dataset supporting the conclusions of this article is available in the OSF repository at https://osf.io/n7e5c/. The experiment was not preregistered.

## Appendix A

### Memory measures 1: Conditional level identification measures

Conditional Level Identification Measures (CLIM) are based on Conditional Source Identification Measures (Bröder & Meiser, 2007; Murnane & Bayen, 1996) in source memory research and are calculated by dividing the number of correct level attributions by the number of hits (i.e., the sum of “high”, “medium”, and “low” responses). For observed response frequencies, see Table 4. For example, to calculate how well participants classified high participation levels, one would use the formula:

$${\text{CLIM}}_{\text{HighParticipation}}=\frac{{\text{High}}_{\text{High}}}{{\text{High}}_{\text{High}}+ {\text{Medium}}_{\text{High}}+ {\text{Low}}_{\text{High}}}=\frac{148}{148+182+111}= .33$$

where: High_High_ = Number of “high” responses for high participation targets (correct responses), Medium_High_ = Number of “medium” responses for high participation targets (incorrect responses), Low_High_ = Number of “low” responses for high participation targets (incorrect responses).

To give another example, these classification-based measures for low competence would be calculated by:

$$\text{CLIM}_{\text{LowCompetence}}=\frac{{\text{Low}}_{\text{Low}}}{{\text{High}}_{\text{Low}}+ {\text{Medium}}_{\text{Low}}+ {\text{Low}}_{\text{Low}}}=\frac{129}{80+203+129}=.31$$

where: High_Low_ = Number of “high” responses for low competence targets (incorrect responses), Medium_High_ = Number of “medium” responses for low competence targets (incorrect responses), Low_High_ = Number of “low” responses for low competence targets (correct responses).

Mean CLIM scores are depicted in Fig. 4a. Note that this process is done on an individual level and such scores can be obtained for each participant, allowing to test for statistical relationships with other measures (such as partner selection ratings in the partner model formation phase). While classification-based CLIM scores can serve as an indicator of memory performance, such measures also sometimes confound memory and guessing (see next section).

**Table 4:** Aggregated response frequencies in the partner model retrieval phase

Original level	Participants’ responses							
	Participation				Competence			
	»High«	»Medium«	»Low«	»New«	»High«	»Medium«	»Low«	»New«
High	148	182	111	75	178	165	89	72
Medium	97	197	137	85	103	204	90	107
Low	75	168	197	76	80	203	129	92
New	16	68	60	630	28	63	37	628

Original level = the level of the potential learning partner in the partner model formation phase. Participants’ responses were given in the partner model retrieval phase

### Memory measures 2: multinomial processing tree models

Multinomial processing tree (MPT) models (for a review, see Erdfelder et al., 2009) offer a remedy for the problem that certain measures confound memory and guessing. For instance, one can notice in Table 4 that when participants do not remember the level of the partner, the response “medium” is given most of the times. Take, for example, the participation condition: when participants did not remember the level of partners with original high levels (first line), the answer “medium” was given 182 times (even more so than the correct answer high, which was given 148 times) and “low” was given 111 times. Additionally, when participants forgot the low level of the learning partner, the incorrect answer “medium” was given 168 times while the incorrect answer “high” was given 75 times. This illustrates that sometimes, certain guessing tendencies (here: to often guess “medium”) can lead to inaccurate estimations of memory (here: a potential overestimation of memory for medium levels).

MPT models have been used in several previous studies examining memory for social information with three information classes (e.g., memory for trustworthy, neutral, and cheater behavior, Buchner et al., 2009; Hechler et al., 2016). Based on observed response frequencies (Table 4), these models estimate separate probabilities for certain cognitive processes, e.g., “memory for group awareness (GA) level” or “guessing that the level was medium”. The model adapted for our purposes (Fig. 3) contains 12 parameters for each experimental condition (*D*_High_, *D*_Medium_, *D*_Low_, *D*_New_, *d*_High_, *d*_Medium_, *d*_Low_, *a*_Medium_, *a*_High_, *g*_Medium_, *g*_High_, *b*). Each parameter represents a probability of a certain cognitive process, ranging from 0 to 1. We used two sets of the model (Fig. 3), one set for each experimental condition in which the levels represented the past participation or competence of the potential learning partners.

To illustrate, the first tree of the model (Fig. 3, *i* = high) for the competence condition depicts a situation in the partner model retrieval phase in which participants must judge the level of a presented partner who was originally associated with a high level of competence. Several cognitive processes (represented by letters along the lines) lead to different possible classifications (rectangles on the right side). Participants could answer correctly that this person was associated with a high level, or incorrectly that this person was associated with a medium level, low level, or that this person was not presented before in the partner model formation phase (“new”). With the probability *D*_High_, the partner is correctly recognized as old (i.e., that the partner was presented before in the partner model formation phase) and with the probability *d*_High_, the level of the partner (high) is correctly remembered. However, with the probability 1 − *d*_High_, the level cannot be remembered and participants have to guess the level. With the probability (1 − *a*_Medium_) × *a*_High_, the correct level “high” will be guessed, while the incorrect answer “medium” will be given with the probability *a*_Medium_ and “low” with the probability (1 − *a*_Medium_) × (1 − *a*_High_). If the partner cannot be remembered as old (i.e., that the partner was presented before), they can be guessed to be old with the probability *b* and consequently, the level has to be guessed. Here, when the partner was guessed to be old, the guessing processes follow an analog principle, but are represented by parameters *g* instead of *a*. If the partner is not guessed to be old, participants will incorrectly classify the partner as a new one with the probability 1 − *b*. Consequently, the participant might give the answer “three stars (high competence)” based on correct memory processes (*D*_High_ × *d*_High_) or based on different guessing processes also leading to the correct answer, for example *D*_High_ × (1 − *d*_High_) × (1 − *a*_Medium_) × *a*_High_. The second (*i* = medium), third (*i* = low), and fourth (new partners) trees follow an analogue logic. As new partners were not associated with a certain GA level before, the processing tree for new partners does not contain a *d*-parameter. This example illustrates that (combinations of) several cognitive processes can result in the same response categories, and consequently, that a right answer in the memory test does not necessarily mean that the level was remembered. All parameter estimates of our model are given in Table 5. Hypotheses are tested by imposing additional parameter restrictions on the base model. For example, the hypothesis that high competence levels should be remembered better than medium competence levels translates to the restriction *d*_High_ = *d*_Medium_ in the competence conditions. Whether such restrictions fit the data is expressed in the test statistic Δ*G*^2^ and a significant increase in model misfit indicates that additional restrictions are not appropriate.

**Table 5:** Multinomial processing tree model-based parameter estimates for different memory and guessing processes in both conditions

	Participation		Competence	
*D*_High_	.69	(0.04)	.74	(0.04)
*D*_Medium_ = *D*_New_	.65	(0.02)	.62	(0.03)
*D*_Low_	.69	(0.04)	.67	(0.04)
*d*_High_	.18	(0.05)	.27	(0.04)
*d*_Medium_	.00	(0.08)	.00	(0.09)
*d*_Low_	.25	(0.05)	.11	(0.05)
*a*_Medium_	.47	(0.03)	.52	(0.03)
*a*_High_	.48	(0.04)	.50	(0.04)
*g*_Medium_	.47	(0.04)	.49	(0.04)
*g*_High_	.21	(0.05)	.44	(0.06)
*b*	.53	(0.03)	.44	(0.03)

*D* = probabilities of recognizing a learning partner as already presented or as new (old-new-recognition). *d* = probabilities of remembering the specific level (high, medium, low) of participation or competence of a partner. *a*_Medium_ = the probability of guessing the level “medium”. *a*_High_ = the probability of guessing the level “high”, given it was not guessed to be medium. *g*_Medium/High_ = level guessing probabilities of unrecognized partners. *b* = probability of guessing that a presented partner was presented before. Values in parentheses represent standard errors

## Abbreviations

CLIM: Conditional level identification measure
GA: Group awareness
M: Mean
MPT: Multinomial processing tree
SCO: Social comparison orientation
SEM: Standard error of the mean

## Footnotes

1 Social Comparison Orientation (SCO) and Social Comparison Motives (SCM) describe related but distinct constructs. While SCO reflects a general, habitual tendency to engage in social comparisons, SCM capture more deliberate, strategic reasons for comparison (e.g., comparing for self-enhancement or self-improvement). We decided not to include SCM in our main analyses, as this would have required the introduction of additional theoretical distinctions in the introduction, moderated analyses (see Ray et al., 2017), and detailed discussions. To maintain conceptual clarity and analytic focus, we concentrated on the more general and widely established construct of SCO.

2 Note, however, that MPT models do not always estimate parameters on group levels. In our study, we used a frequentist statistical framework with a complete-pooling approach to estimate memory parameters. Bayesian partial pooling approaches can estimate individual-level parameters and are particularly suitable when the goal is to examine associations between (memory) parameters and other variables (for an overview and comparison of methods, see Singmann et al., 2024). However, these models can require larger sample sizes and more responses per participant to yield stable estimates (Schmidt et al., 2023) and can thus be underpowered in limited sample sizes. In our experiment, we were mainly interested in specific memory differences (*H2* and *H3*). The power to detect differences in corresponding parameters (*d*_High_, *d*_Medium_, *d*_Low_) was already limited, as the corresponding trees did not have many observations, the parameters only occured on single branches of the model, and were conditional (see also Schmidt et al., 2023). Given the limited sample size and the main objective to analyze specific memory differences, we opted for a frequentist framework and complete-pooling approach to maximize statistical power.
